# Characterization of six new complete mitochondrial genomes of Chiasmodontidae (Scombriformes, Percomorpha) and considerations about the phylogenetic relationships of the family

**DOI:** 10.5808/gi.22041

**Published:** 2023-03-31

**Authors:** Igor Henrique Rodrigues-Oliveira, Rubens Pasa, Fabiano Bezerra Menegidio, Karine Frehner Kavalco

**Affiliations:** 1 Institute of Biological Sciences, Federal University of Minas Gerais, Belo Horizonte 31270-901, Brazil; 2 Laboratory of Ecological and Evolutionary Genetics, Federal University of Viçosa, Rio Paranaíba 38810-000, Brazil; 3 Laboratory of Bioinformatics and Genomics, Federal University of Viçosa, Rio Paranaíba 38810-000, Brazil; 4 Technological Research Center, University of Mogi das Cruzes, Mogi das Cruzes 08780-911, Brazil; 5 Integrated Biotechnology Center, University of Mogi das Cruzes, Mogi das Cruzes 08780-911, Brazil

**Keywords:** Cretaceous-Paleogene extinction event, mitogenome, Pelagiaria, phylogeny, swallowers fishes

## Abstract

The fishes of the Chiasmodontidae family, known as swallower fishes, are species adapted to live in deep seas. Several studies have shown the proximity of this family to Tetragonuridae and Amarsipidae. However, the phylogenetic position of this clade related to other Pelagiaria groups remains uncertain even when phylogenomic studies are employed. Since the low number of published mitogenomes, our study aimed to assemble six new mitochondrial genomes of Chiasmodontidae from database libraries to expand the discussion regarding the phylogeny of this group within Scombriformes. As expected, the composition and organization of mitogenomes were stable among the analyzed species, although we detected repetitive sequences in the D-loop of species of the genus *Kali* not seen in *Chiasmodon*, *Dysalotus*, and *Pseudoscopelus*. Our phylogeny incorporating 51 mitogenomes from several families of Scombriformes, including nine chiasmodontids, recovered interfamilial relationships well established in previous studies, including a clade containing Chiasmodontidae, Amarsipidae, and Tetragonuridae. However, phylogenetic relationships between larger clades remain unclear, with disagreements between different phylogenomic studies. We argue that such inconsistencies are not only due to biases and limitations in the data but mainly to complex biological events in the adaptive irradiation of Scombriformes after the Cretaceous-Paleogene extinction event.

## Introduction

The Chiasmodontidae family comprises 36 species of marine fishes belonging to four genera with a worldwide distribution [1]. This group, traditionally known as swallowers fishes or snake tooth fishes, have mesopelagic and bathypelagic habits living below 200 m depth [2]. As a result of the selective pressures of the environment with few food resources, these fish are known for their small size [2] and expandable stomachs, allowing the ingestion of prey much larger than the animal itself [3].

Interfamilial relationships of Chiasmodontidae with other groups are uncertain. Different phylogenetic hypotheses were postulated in recent years [4-8]. Miya et al. [4] included Chiasmodontidae and 14 other families in the Pelagiaria clade, highlighting the high support on the monophyly of the group. In addition, they argued that Amarsipidae and other Stromateoidei belong to the same clade.

Like Chiasmodontidae and other Pelagiaria groups, Stromateoidei was part of the order Perciformes in the past, until Nelson et al. [9] included Stromateoidei and Scombroidei in a new order called Scombriformes. Studies before Nelson et al. [9] had already shown that both Stromateoidei and Scombroidei suborders did not correspond to natural groupings [4-5,10,11]. Considering the phylogenetic relationships of most families traditionally allocated to these suborders with other groups within the Pelagiaria clade, Betancur et al. [5] included Chiasmodontidae and seven other Pelagiaria families in Scombriformes. Although Betancur et al. [5] recognized that interfamilial relationships of Scombriformes were uncertain, genomic studies have demonstrated a strong phylogenetic relationship between Chiasmodontidae with Tetragonuridae [4-8,12] and Amarsipidae [7,8].

To date, only the mitochondrial genomes of *Chiasmodon harteli* (unpublished data), *Chiasmodon asper*, *Dysalotus alcocki*, and *Kali indica* of the Chiamodontidae family have been published [4]. However, only *K. indica*, *D. alcoki*, and *C. harteli* have their mitogenomes deposited in the GenBank (the accession number provided for *C. asper* corresponds to the mitogenome of *Champsodon* cf. *snyderi* in the NCBI). This number is very small compared to the related family Scombridae, for example, which has dozens of mitogenomes deposited in the NCBI. In this way, our work aimed to assemble six new mitochondrial genomes for the group, expand the knowledge of the evolution and mitochondrial diversity of the family and provide new perspectives on their phylogenetic relationships.

## Methods

We obtained raw library data of *Chiasmodon niger* (SRX10444742), *Dysalotus oligoscolus* (SRX7174474), *Kali macrodon* (SRX7174492), *Kali macrura* (SRX7174493), *Kali normani* (SRX7174494), and *Pseudoscopelus astronesthidens* (SRX7174526) from the Sequence Reads Archive (SRA) NCBI (Table 1).

While the raw data from *C. niger* were obtained by targeted enrichment of single copy exons (exon capture) [7], the other data were obtained by target capture of ultraconserved nuclear elements (UCEs) [12]. Although both types of sequencing do not include complete genome sequences, sequence capture is not 100% accurate, and due to the presence of off-target sequences, it is possible to assemble mitogenomes from both exome and UCEs data [13,14].

We imported the raw data into the Galaxy Europe platform [15] and used NOVOplasty v4.3.1 [16] to assemble the mitochondrial genomes by the "*de novo*" method, ie without reference. Following the instructions of the NOVOplasty software (available at: https://github.com/ndierckx/NOVOPlasty) we did not filter or quality trim the reads and used the raw genome dataset. As a seed, we used the complete mitogenome of *K. indica* (NC_022488.1) in the *K. macrodon* and *K. normani* assembling, and for the other species, we used the corresponding COI mitochondrial gene sequences deposited in GenBank (Supplementary Material 1).

We annotated all mitogenomes using MitoAnnotator [17] available on the MitoFish server (http://mitofish.aori.u-tokyo.ac.jp). We used the BLAST Ring Image Generator (BRIG) [18] to perform a comparative BLAST analysis of available Chiasmodontidae mitogenomes (literature and our assemblies) against our *C. niger* mitogenome assembly. We used the Tandem Repeats Finder [19] to visualize possible tandem repeat sequences in the mitogenomes.

To perform the phylogenetic analyses, we manually extracted the sequences of the 13 protein-coding genes (PCGs) from our assemblies and added to the dataset the same genes from the mitogenomes of *K. indica* (NC_022488.1), *D. alcoki* (NC_022482.1), *C. harteli* (AP012975.1), 42 mitogenomes from the other 15 families of Scombriformes (Supplementary Material 2), and *Aeoliscus strigatus* (Syngnantiformes) as an outgroup (NC_010270.1). We aligned the sequences with Muscle [20] in Mega X software [21] and concatenated the alignments in SequenceMatrix v1.8 [22]. We constructed the phylogeny by the maximum likelihood method in IqTree v2.1.2 software [23] using 1,000 ultrafast bootstrap replications and using the evolutionary model TVM+F+R6 estimated by the ModelFinder [24] of IqTree.

The mitochondrial genomes have been deposited in GenBank under the following accession numbers: *C. niger*, ON831389; *D. oligoscolus*, ON831390; *K. macrodon*, ON831391; *K. macrura*, ON831392; *K. normani*, ON831393 and *P. astronesthidens*, ON831394.

## Results and Discussion

**Fig. 1.**
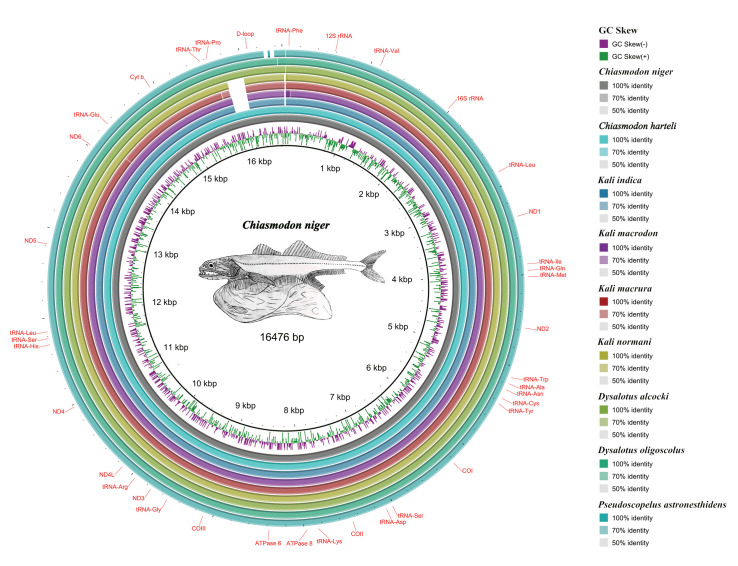
Comparative mitogenomics analysis of all the nine chiasmodontid fishes against a reference (*Chiasmodon niger*), generated by BLAST Ring Image Generator (BRIG). Gaps in rings correspond to regions with <50% identity to the reference sequence (BLAST comparison).

As expected, all mitogenomes showed the same arrangement and gene content (Fig. 1), with 13 PCGs, 22 tRNAs, two rRNAs, and a control region (D-loop), with all PCGs (except *ND6*) in the heavy chain and eight tRNAs in the light chain, as has been observed in most vertebrate mitogenomes, including teleosts, described [25].

We deposited all mitogenomes in GenBank (Supplementary Material 1). Mitogenomes ranged from 16,468 bp in *P. astronesthidens* to 16,627 bp in *K. macrodon* and *K. normani*. We found tandem repeats in the D-loop of all three species of the genus *Kali*, with three repeating sequences in *K. macrura* and five in *K. macrodon* and *K. normani* (Supplementary Material 3), but we did not find repeat regions in the mitogenomes of *C. niger*, *D. oligoscolus*, and *P. astronesthidens*. Repetitive sequences in the D-loop shared between species of the same genus have already been reported for fishes from the tribe Gymnocharacini and may play a key role in the study of the evolutionary history of these groups [26].

**Fig. 2.**
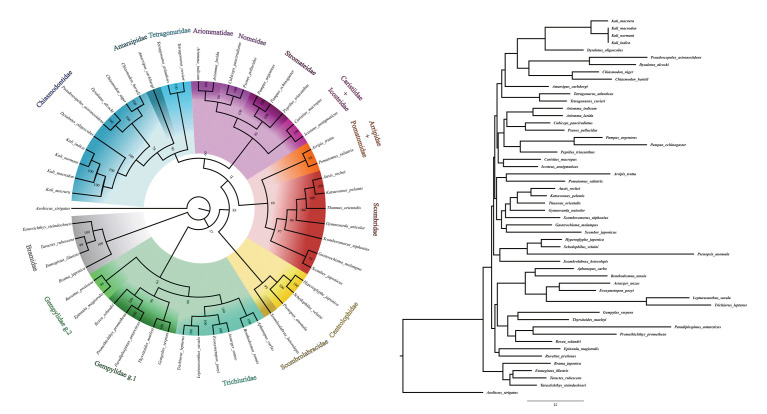
The phylogenetic tree of Chiasmodontidae mitogenomes and other 43 species available in GenBank. The bootstrap values were indicated in each branch of the tree. *Aeoliscus strigatus* was selected as an outgroup.

Among the intrafamilial phylogenetic relationships observed for Chiasmodontidae, all species of the genera *Chiasmodon* and *Kali* formed monophyletic groups, and the two *Dysalotus* species used did not cluster (Fig. 2), rather than observed in previous phylogenomic studies [8]. Chiasmodontidae formed a clade with Tetragonuridae and Amarsipidae, corroborating previous phylogenomics and mitogenomics studies [4,7,8,12]. As observed in previous studies [4,6-8,12], other interfamilial phylogenetic relationships recovered in this work were: Stromateidae as a sister group of Nomeidae + Ariommatidae and Trichiuridae as a sister group of Gempylidae (Fig. 2). These clusters correspond to clades A (Stromateidae, Ariommatidae and Nomeidae), C (Chiasmodontidae, Tetragonuridae and Amarsipidae) and partially B (Gempylidae and Trichiuridae, but not Scombrolabracidae) described by Arcila et al. [7].

In the same way Miya et al. [4] and Campbell et al. [6], but unlike Friedman et al. [12], Arcila et al. [7], and Harrington et al. [8], we recovered Caristiidae as a sister group to Icosteidae and Pomatomidae as a sister group to Arripidae. As well as the mitogenomic analysis by Miya et al. [4] we also recovered Gempylidae as a paraphyletic group related to Trichiuridae, and Centrolophidae as a sister group of Scombrolabracidae (Fig. 2). However, the relationships between larger clades containing two or more closely related families remain uncertain and discordant among the different phylogenetic analyzes cited here.

The increase in the number of published mitogenomes associated with the stable organization of this genome among vertebrates, the improvement of assembly techniques, and its maternal nature without recombination make the mitochondrial genome a tool with great potential to solve taxonomic problems and phylogenetic relationships [26,27]. Furthermore, as all mitochondrial genes are linked on the same chromosome, they have a very similar phylogenetic signal and share a unique phylogenetic history, allowing them to be used concatenated and partitioned in phylogenetic analyses [28]. However, incongruities between nuclear and mitochondrial data are frequently reported in the literature. These inconsistencies may be related to data biases and limitations such as saturation, incomplete lineage sampling, differences in taxa sampling, gene partitioning, and other phylogenetic methods employed [27,29-31]. Important biological factors may underlie these divergences, such as past events of hybridizations, selection, and complex biogeographic events with secondary contact of allopatric populations and replacement of mitochondria [29,31,32].

Since the Scombriformes experienced rapid adaptive radiation after the Cretaceous-Paleogene mass extinction [4,12] the high degree of uncertainty reported in the phylogenetic relationships of the group is natural [6,7]. Although some interfamilial phylogenetic relationships, such as Chiasmodontidae with Tetragonuridae and Amarsipidae, are strongly supported, major clade relationships remain uncertain even when phylogenomic approaches are employed, which may result from complex biological events during post-mass extinction adaptive irradiation, as hybrids between lineages, like the radiation of placental mammals [33]. In this way, the phylogenetic representation through two-dimensional trees may not be the best way to illustrate the evolutionary history of the Scombriformes, by “hiding” such events. Finally, we strongly suggest that future phylogenetic analyzes of the group not only increase the amount of data used but also incorporate phylogenetic network analyses. Compared to conventional phylogenetic trees, phylogenetic networks can visualize in a single image conflict between different phylogenetic hypotheses using crosslinks between branches. This type of analysis can be used when complex biological events, called reticulated events, such as hybridizations, recombination, horizontal gene transfer, duplications or gene loss are suspected [33,34].

## Figures and Tables

**Table 1.** Raw genomic data obtained from the NCBI for each species

Species	BioProject ID	SRA ID	Raw reads
*Chiasmodon niger*	PRJNA644198	SRX10444742	510.7M
*Dysalotus oligoscolus*	PRJNA561597	SRX7174474	326.2M
*Kali macrodon*	PRJNA561597	SRX7174492	330.9M
*Kali macrura*	PRJNA561597	SRX7174493	743.2M
*Kali normani*	PRJNA561597	SRX7174494	142.1M
*Pseudoscopelus astronesthidens*	PRJNA561597	SRX7174526	420.6M

## Supplementary Materials

Supplementary Table is available from the Figshare repository (https://doi.org/10.6084/m9.figshare.19952939). Raw sequencing reads are available at NCBI Sequence Read Archive (SRA), under BioProject number PRJNA561597 and PRJNA644198.
